# Yankeedom out Yankied

**Published:** 1854-05

**Authors:** 


We have a brother who edits a newspaper in Texas, called the Bonham Advertiser. In his issue for March 1, 1854, we find the following paragraph. It is quite possible that our readers may see in it some family likeness to ourselves. Probably Holloway's Pills and Ointment will sell well in Fannin county hereafter: H.

" *Yankeedom Out Yankied.*—By the last mail we received the following modest and obliging epistle, all the way from London, dated '244 Strand, 3d January, 1854:'

'DEAR SIR,—I beg leave to refer you to my letter of 2d August, 1853, containing a proposition for the insertion of my advertisements and paragraphs in your paper and to which I have had no reply. Inferring from your silence that the terms offered were lower than you were willing to accept, I now beg leave to submit the following proposal to your consideration.

I shall be happy to pay you the sum of twenty-four dollars per annum for the insertion of one of the inclosed advertisements, with one of the paragraphs, in every issue of your paper, the blocks, which will be supplied free of charge, on application to my agent for your state, at Galveston, to be used as headings, the agents, names at foot, and the paragraphs to appear among the miscellaneous news, fresh matter every week.

'You must also forward me, free of charge and U. S. postage, a copy of every issue of your paper in which my advertisement appears, for my file and news-room. This is a most important point, and one which must have your punctual attention.

'I assure you that I will at all times do all that lays in my power to promote the interests of your paper, and you are at liberty to announce that a file of the same is kept at my establishment, and I have no objection to receive subscriptions, or orders for advertisements, if I can render you a service by so doing.

'Hoping to receive an early answer, I remain, dear sir, yours faithfully,

'THOMAS HOLLOWAY.'

"Accompanying this letter were copies of two advertisements — one of a miraculous ointment, and one of an omnipotent pill, — an article on making fortunes by advertising, — and fifty-two instances of remarkable cures performed four thousand miles distant from this market, in which Mr. Holloway desires our endorsement of his medicines. At the head of the list of cures is the following adroit Yankeeism:

'N. B. — The compositor will kindly oblige the proprietor by inserting, during the winter season, or wet weather, the paragraphs in this list relating to colds, coughs, asthmas, and rheumatism; and in the summer and fruit season, those for diarrhœa, bilious and bowel complaints, indigestion, &c.; and about the autumn, and the end of spring, the cases that apply to dropsy and skin diseases.'

"This is making a commodity of disease with a closeness of calculation beyond the capacity of John Bull. Ten to one, Mr. Holloway is Yankee turned Cockney.

"And now, Mr. H., after thanking you for your offer to extend our already enormous circulation in England, we beg leave to inform you that we will not advertise your nostrums at any price. We believe you to be a quack, and of that kind of cattle we have plenty at home. We may advertise for them, when they ask no more of us than to publish what they sign; but this paragraph business makes us a party to what we deem a deliberate swindle. It will not be worth your while to address us again on this subject. You have *this* number of our paper, post-paid; you can have the remaining fifty-one for your file and news-room by remitting to us the sum total of two dollars and twenty-five cents."

